# Echocardiographic Parameters as Predictors for the Efficiency of Resynchronization Therapy in Patients with Dilated Cardiomyopathy and HFrEF

**DOI:** 10.3390/diagnostics12010035

**Published:** 2021-12-24

**Authors:** Silvius-Alexandru Pescariu, Raluca Şoşdean, Cristina Tudoran, Adina Ionac, Gheorghe Nicusor Pop, Romulus Zorin Timar, Sorin Pescariu, Mariana Tudoran

**Affiliations:** 1 Department VI, Discipline of Cardiology, University of Medicine and Pharmacy “Victor Babes” Timisoara, E. Murgu Square, Nr. 2, 300041 Timisoara, Romania; pescariu.alexandru@umft.ro (S.-A.P.); ionac.adina@umft.ro (A.I.); pop.nicusor@umft.ro (G.N.P.); pescariu.sorin@umft.ro (S.P.); 2 Cardiology Clinic, Institute of Cardiovascular Medicine Timişoara, 13A, Gheorghe Adam Street, 300310 Timisoara, Romania; 3 Research Center for Cardiovascular Diseases, Institute of Cardiovascular Diseases, 300310 Timisoara, Romania; 4 Doctoral School, “Victor Babes” University of Medicine and Pharmacy Timisoara, E. Murgu Square, Nr. 2, 300041 Timisoara, Romania; 5 Department VII, Internal Medicine II, Discipline of Cardiology, University of Medicine and Pharmacy “Victor Babes” Timisoara, E. Murgu Square, Nr. 2, 300041 Timisoara, Romania; timar.romulu@umft.ro (R.Z.T.); tudoran.mariana@umft.ro (M.T.); 6 Center of Molecular Research in Nephrology and Vascular Disease, Faculty of Medicine, University of Medicine and Pharmacy “Victor Babes” Timisoara, E. Murgu Square, Nr. 2, 300041 Timisoara, Romania; 7 County Emergency Hospital, L. Rebreanu Str., Nr. 156, 300723 Timisoara, Romania

**Keywords:** transthoracic echocardiography, longitudinal strain, sensing parameters, pacing parameters

## Abstract

Cardiac resynchronization therapy (CRT) represents an increasingly recommended solution to alleviate symptomatology and improve the quality of life in individuals with dilated cardiomyopathy (DCM) and heart failure (HF) with reduced ejection fraction (HFrEF) who remain symptomatic despite optimal medical therapy (OMT). However, this therapy does have the desired results all cases, in that sometimes low sensing and high voltage stimulation are needed to obtain some degree of resynchronization, even in the case of perfectly placed cardiac pacing leads. Our study aims to identify whether there is a relationship between several transthoracic echocardiographic (TTE) parameters characterizing left ventricular (LV) performance, especially strain results, and sensing and pacing parameters. Between 2020–2021, CRT was performed to treat persistent symptoms in 48 patients with a mean age of 64 (53.25–70) years, who were diagnosed with DCM and HFrEF, and who were still symptomatic despite OMT. We documented statistically significant correlations between global longitudinal strain, posterolateral strain, and ejection fraction and LV sensing (r = 0.65, 0.469, and 0.534, respectively, *p* < 0.001) and LV pacing parameters (r = −0.567, −0.555, and −0.363, respectively, *p* < 0.001). Modern imaging techniques, such as TTE with cardiac strain, are contributing to the evaluation of patients with HFrEF, increasing the chances of CRT success, and allowing physicians to anticipate and plan for case management.

## 1. Introduction

Dilated cardiomyopathy (DCM) carries a significant burden for the patients and for the health care system and physicians as well. As the disease progresses, treatment options involving medication become limited as heart failure (HF) continues to worsen, severely limiting the patient’s ability to perform even the smallest of daily tasks [1]. Fortunately, modern imaging techniques provide superior insight into the extent of the disease. Speckle tracking with cardiac strain is a modern imaging technique that analyses in detail the segmental shortening of the ventricular myocardium [2,3]. These ultrasonographic investigations are vital in assessing the severity of the disease as well as its progression. As the disease progresses, an increasing amount of left ventricular (LV) intramyocardial fibrosis is detected. This happens independent of the etiology of HF, although in ischemic patients the amount of fibrosis and scar burden is much more evident and detected earlier in the disease evolution [4]. This aspect may interfere with the pacing and sensing thresholds of a cardiac resynchronization therapy (CRT) system, by altering them. Currently, the best way to evaluate intramyocardial fibrosis is by use of cardiac magnetic resonance imaging (cMRI) with late gadolinium enhancement for scar evaluation and T1 mapping for diffuse fibrosis. This imaging modality is not always available and is time consuming; however, the evaluation of longitudinal strain by the echocardiographic speckle tracking technique has concordant results and is more widely available [4,5]. Longitudinal strain is more reproducible as compared to radial and circumferential strain, and it evaluates the contraction of the longitudinal fibers, which are the first to be affected [4,6,7]. In terms of current treatment options, currently, interventional cardiology comes to the aid of these patients employing CRT [8,9]. Patients with left bundle branch block (LBBB) and significant systolic dysfunction, expressed by HF with reduced ejection fraction (HFrEF), can benefit from this course of treatment according to current guidelines [1,10]. CRT has been proven to be superior to conventional medical treatment by decreasing mortality and morbidity among patients who suffer from DCM, be it of ischemic or idiopathic etiology [11,12]. However, CRT poses a set of particular challenges, being less successful in certain patients who have high pacing thresholds which need constant adjustment and intermediate sensing values, despite the optimal lead placement. High acute pacing thresholds are especially detrimental since a higher voltage for pacing translates into a higher rate of pulse generator battery discharge, leading to an important decrease in the battery’s longevity, which consequently leads to the need for earlier generator replacement [13,14,15]. With pacing thresholds being known to vary, sometimes failure-to-capture can occur over time, requiring even further amplification of the stimulation voltage. Interventions for replacing the pulse generator carry some degree of risk for the patient as infections of the device pocket are extremely dangerous since they can lead to exteriorization, endocarditis, or even sepsis and are seldom curative without the extraction of the entire pacing system, a difficult maneuver with considerable risks [14,16,17]. Individuals implanted with CRT defibrillator (CRT-D) are especially vulnerable in the event of discharged generators, since they require the additional protection that the defibrillator offers. As a result of the thresholds increasing over time, a failure in the impulse capturing can result in pacing deficits and/or inefficient CRT therapy.

This study aims to investigate whether several TTE parameters characterizing cardiac performance, and especially the strain imaging techniques such as LV global longitudinal strain (LV-GLS) and LV postero-lateral strain (LV-PLS) could be considered as predictors for suboptimal CRT functional parameters, namely low sensing, and high pacing, in patients with DCM and HFrEF.

## 2. Materials and Methods

### 2.1. Study Population

A single cohort of 48 patients suffering from DCM and HFrEF, who were implanted during January 2020 and June 2021 with three-lead pacemaker CRT (CRT-P) and three-lead intracardiac defibrillator (CRT-D) devices at the Institute of Cardiovascular Diseases from Timisoara, Romania, represent our study population. All patients were symptomatic despite optimal medical therapy (OMT), all had LBBB and were implanted with these devices according to current European Society of Cardiology (ESC) guidelines [1,10]. Patients with permanent atrial fibrillation who received biventricular pacemakers (CRT-P) were also included in the study as the main goal was to evaluate the impact of global and focal longitudinal strain on pacing parameters. All patients were evaluated by TTE before the implantation procedure.

All patients signed the standardized informed consent form required by the Health Authority of Romania at hospital admission, and their data were anonymized before data collection. The Ethics Committee of the Institute of Cardiovascular Diseases, Timisoara approved this study, Nr. 4052/19.06.2020.

### 2.2. Echocardiography

A complete bidimensional TTE evaluation was performed by a single blinded cardiologist, with advanced training in echocardiography, in all patients approximately 1 day before CRT intervention, using the General Electric VIVID E95 ultrasound system (GEMS Ultrasound, Horten, Norway). Several parameters reflecting LV (end-diastolic volume, ejection fraction, global and segmental longitudinal strain, volume) and right ventricular (RV, including longitudinal contraction, pulmonary artery systolic pressures) morphological and functional status, as well as atrial morphological status (left atrial volume), were selected to be analyzed for this study.

The left atrial volume (LAV) was measured by planimetry in a biplane manner, in 4 chambers and 2 chambers views, respectively, according to the European Association of Cardiovascular Imaging (EACVI) recommendations [18].

The LV volumes (end-diastolic volume—EDV and end-systolic volume—ESV) were measured by planimetry (tracing the interface between the myocardium and the cavity), also in a biplane manner, in 4 chambers and 2 chambers views, respectively. The LV ejection fraction (LVEF) was measured by using the biplane modified Simpson’s method [3,19].

The longitudinal strain was evaluated by using the speckle tracking technique, in 4, 2, and 3 chamber views. The region of interest (ROI) was automatically rendered, with subsequent manual adjustments to obtain the best delineation of the myocardium to be analyzed. Care was taken not to include the pericardium in the ROI, and thus obtain falsely decreased absolute strain values. The LV was divided into 6 segments to be analyzed-basal, mid-ventricular, and apical, in each view. Three cardiac cycles were recorded for offline analysis in each view, at a frame rate between 40 and 90 frames/s, depending on the heart rate (higher frame rate for higher heart rates). The global longitudinal strain was calculated on the resultant bull’s eye model [2,19,20,21].

The longitudinal contraction of the RV was evaluated by measuring the tricuspid annular plane systolic excursion (TAPSE). This parameter was measured according to the EACVI chamber quantification recommendations, by M-mode, between end-diastole and peak-systole [3,19].

The systolic pulmonary artery pressure (sPAP) was evaluated by measuring the tricuspid regurgitation flow maximal pressure. The estimated right atrial pressure (RAP) was added to this value (5 mmHg if the diameter of the inferior vena cava (IVC) was <2.1 cm with >50% inspiratory collapse, 10 mmHg if the diameter of the IVC was >2.1 cm with >50% inspiratory collapse or the diameter of the IVC was <2.1 cm with <50% inspiratory collapse and 15 mmHg if the diameter of the IVC was >2.1 cm with <50% inspiratory collapse).

### 2.3. Resynchronization Therapy (CRT)

In most patients, the implantation technique consisted of inserting a defibrillator/RV pacing lead into the RV cavity via the cephalic vein which was isolated in the deltopectoral groove. If the vein permitted, the atrial lead was inserted using the same route, if not, the atrial lead was inserted via the subclavian vein after positioning the coronary-sinus (CS) lead. Using the cephalic vein for the insertion of the RV lead allowed us to avoid difficulties in positioning because of lead interactions via the route of the subclavian vein, thus facilitating our control in cannulating the CS and maneuvering the CS lead into the optimal position. Arguably the most difficult step of the procedure is positioning the LV lead. This is mainly due to two anatomical variabilities (1) CS ostium and (2) cardiac veins themselves. Once the CS has been cannulated using the purpose built catheters, contrast is injected to visualize the particular anatomy of the cardiac veins, which presents significant interindividual variety [22]. A guidewire is advanced in order to offer support as well as a pathway for advancing the actual LV lead. The guidewire is then manipulated in such a way that it enters one of the posterolateral branches. These branches allow for stable placement of the LV lead. Despite satisfactory radiological positioning of the lead, sensing and pacing parameters can be substandard, possibly due to an area rich in fibrous tissue. In order to electrically assess the positioning of the leads, sensing and pacing thresholds are evaluated to discern whether it is necessary to adjust the position or possibly relocate the lead to another branch of the CS due to low sensing or high pacing thresholds. After positioning the lead optimally in the CS, by using the cephalic vein for the other two leads, the risk of dislodgement was decreased, as this is an extremely unwanted event that threatens the success of the overall resynchronization therapy. By inserting the defibrillator lead first into the RV cavity, we secured the means of delivering an internal shock if malignant ventricular tachycardia arises during the procedure. This measure is especially important in patients with previously diagnosed ventricular tachycardia who benefited from implantable cardiac defibrillator therapy for secondary prevention.

We consider ideal functioning parameters for pacing around 1 ± 0.5 V for the RV and right atrium leads and around 2 ± 0.5 V for the CS lead with ideal sensing parameters exceeding 10 mV in the case of the CS and RV lead and over 3 mV for the right atrium lead.

### 2.4. Statistical Methods

We employed the Shapiro–Wilk test to evaluate the distribution of numeric variables. Numeric variables were presented as the median and interquartile range (IQR), and categorical variables were presented as frequency and percentages. We employed Wilcoxon test to compare the QRS duration before and after CRT implantation. To evaluate the association between LV sensing/pacing and different echocardiographic parameters we used the Spearman correlation test. To assess the independent factors that influenced the LV sensing/pacing we built several multivariate linear regression models. In the final regression equations, the predictors were accepted according to a repeated backward-stepwise algorithm (inclusion criteria *p* < 0.05, exclusion criteria *p* > 0.10) so as to obtain the most appropriate theoretical model that fit the collected data. The quality of the model was described using the accuracy of prediction and R squared. Data analysis was performed using the Statistical Package for the Social Sciences v.26 (SPSS, Chicago, IL, USA). A *p*-value < 0.05 was considered statistically significant.

## 3. Results

This study included 48 patients with DCM, aged between 39 and 73 years, with a median of 64 (53.25–70) years. Thirty-seven of them were men (77%) and 11 women (23%). Referring to the etiology, 37 had idiopathic DCM (64.6%) and 17 ischemic forms (35.4%). According to their symptomatology, most patients were in class NYHA III (47.9%), followed by class II (41.6%) and class IV (10.4%). The characteristics of the study population, as well as their therapy, are presented in Table 1 and their initial electrocardiographic and TTE parameters in Table 2.

All patients were treated according to the ESC guidelines [1] with OMT according to indications, associated diseases, and side effects, with doses adjusted according to the individual tolerance, Table 1. All of them underwent a detailed TTE examination before the CRT implantation, and their echocardiographic, but also the electrocardiographic parameters before and after the implantation are presented in Table 2.

We observed a statistically significant shorter QRS duration after the CRT implantation (*p* < 0.001, Wilcoxon test). The median values of sensing and pacing parameters of CRT’ devices are presented in Table 3.

By analyzing the existence of statistically significant correlations between several echocardiographic results and LV sensing and pacing, we identified that the most significant ones were evidenced between the strain parameters, followed by the LVEF and LAV both for LV sensing, but especially for LV pacing, Table 4. We also observed a weak, but still statistically significant correlation between sPAP and RV pacing (r = 0.286, *p* = 0.049).

To determine which independent factors, namely the LV sensing and LV pacing thresholds, could be predicted in our study group we employed a multivariate linear regression model. We used the forward stepwise method, and the best models were selected based on Akaike information criteria (AIC). We included in our models parameters such as age, sex, echocardiographic parameters, and patients’ comorbidities.

The regression equation for the LV sensing proved to be adequate for the model, explaining 72.1% of the LV sensing variance (R^2^ = 0.721), the details being presented in Table 5. The LV sensing threshold increased directly proportional with the elevation of LV-GLS and LAV values. The presence of ventricular tachycardia or diabetes mellitus decrease the LV sensing threshold. Additionally, more advanced stages of HF (NYHA) decrease the LV sensing value.

Referring to the LV pacing, the model presented in Table 6 explains 44.6% of its variance (R^2^ = 0.446). Lower values of LV-GLS, RV-GLS, and TAPSE increase the LV pacing threshold, as well as the presence of diabetes mellitus.

## 4. Discussion

According to the newest ESC guidelines, CRT represents a Class I, Level A recommendation for the treatment of HFrEF, with LVEF under 35%, regardless of the NYHA class, in patients who are still symptomatic, despite OMT, to reduce morbidity and mortality, and especially, to alleviate their complaints and to improve their quality of life [1,10,23]. There is research that predicates that the benefits of this therapy are still underutilized [9,24]. All patients included in this study had an indication of Class I for CRT implantation and were referred for this therapy [1,10].

As they were evaluated before the procedure, a comprehensive TTE exam, followed by strain imaging was performed to characterize LV performance, to observe the severity of kinesis abnormalities, and the existence of mechanical asynchronism [25,26]. The results illustrated that ultrasound strain parameters, global and focal as well, can indeed be linked with sensing and stimulation thresholds. As well as finding LV-GLS relevant, we also found focal, LV-PLS to be just as relevant as it evaluates the strain in the regions where the coronary sinus lead will be positioned, which is vital to a successful CRT. As all patients had undergone strain echocardiography before implantation, the results from this noninvasive investigation can help us to guide the interventional cardiologist in programming the device after the implantation has taken place. It is relevant as well as during the procedure when the interventionist is influenced by the acute sensing threshold. Acute sensing thresholds are important in regard to making intraoperative decisions towards positioning the leads, as interventionists seek the optimal position, which usually involves achieving the highest sensing value as this is a marker of how well the electrodes on the lead receive the electrical action potential generated by the myocardium. Pacing thresholds are an even more valuable parameter to predict [15,25,27], as the success of CRT depends on the ability to achieve successful LV and RV pacing. Without efficient impulse captures on either lead, the stimulation will mainly be achieved by just one of the ventricular leads, thus possibly decreasing the ejection fraction even further as well as further altering intra- and interventricular synchronism, consequently having possible effects on the LV-GLS [13,23,28]. Fortunately, the efficiency of CRT therapy can also be evaluated by the simpler means of the surface ECG, with the end optimal result being the reduction in the duration of the QRS complex. The modern CRT devices and leads we use in our patients, mainly manufactured by Biotronik (Berlin, Germany), offer an array of possible programming polarities. By modifying the combination of electrodes, the electrical impulse travels between, we achieve, in most patients, significant reductions in the QRS complex duration, especially in those with significantly prolonged ORS complexes before the procedure [25,29,30].

As debated in the medical literature [23,24,29,31], CRT is one of the most effective therapeutic possibilities for the treatment of patients with HFrEF, which still remains widely underutilized, with only approximately one-third of eligible patients receiving a timely referral for this procedure [9,10,24]. An optimized collaborative work between the cardiologists responsible for the clinical care and evaluation of those patients with the interventional cardiologist could improve the management of patients with HFrEF [1,10,24,30], even in cases where it seems more difficult to obtains the optimal results.

Our results are explained by the fact that GLS (local and global) reflects the fibrosis burden in the myocardium. It is demonstrated that scar tissue does not have any electrical activity therefore no pacing and/or sensing is possible [32,33]. It is recommended to avoid placing the CS lead on scar tissue and also in its immediate proximity, as the scar induces fragmentation of the electrical impulse. Abozguia et al., used a multipolar LV lead in order to counteract the deleterious scar effects, with good results [34]. Mele et al., evaluated the response of patients with CRT with the LV pacing lead placed on, adjacent to and/or remote from a scared segment, considered as therapy responders the patients with at least 10% decrease in LV end-systolic volume at 6 months. They demonstrated that the CRT response depended on the quality of the pacing site underlying tissue in addition to the global LV scar burden [35]. Mendonca Costa et al., also demonstrated that pacing in the proximity of a scar may induce ventricular arrhythmias. Using images from a number of 24 patients with ischemic DCM, they created computational models (patient specific, LV anatomic, scar morphology included) through which they demonstrated an increased repolarization dispersion inducing an arrhythmogenic substrate [36].

In the same manner, even if there is no consistent scar on the LV lead targeted wall, a high amount of diffuse/interstitial or replacement fibrosis explain the altered of the pacing and sensing thresholds. The low response to CRT in these patients is induced, as demonstrated previously by several authors [32,33,34,35], using the fragmentation of the impulse conduction, with a significant increase in conduction delay and prolongation of the QRS length, but also by a possible intermittent loss of LV capture, which is sometimes hard to diagnose just by interrogating the device.

It is true that longitudinal strain evaluates the longitudinal fibers contraction, fibers that are best represented (but not exclusively) in the subendocardial layer [18], and the LV lead is placed in one of the CS branches, most of the time a postero-lateral branch to serve the most delayed contracting wall, which is on the epicardial side of the LV. However, the impulse must travel from the epicardial side to the endocardial side and meet the impulse induced by the RV lead, which travels from the endocardium to the epicardium, in order to obtain a homogenous and faster depolarization and synchronize the contraction. If the impulse is delivered in or in the proximity of a high fibrosis area, its quality will be compromised, as this type of tissue has altered fiber orientation and a reduced electrical conductivity [37].

The main limitation of our study is represented by the small number of patients treated with CRT. The low case numbers were due to the COVID-19 pandemic with admission of non-COVID patients and reduced access to the interventional laboratory. As a consequence of the small patient population, we could not further analyze the impact of other factors, especially of associated diseases, on our results.

## 5. Conclusions

Modern imagistic techniques, such as cardiac strain, are contributing to the evaluation of patients with heart failure with HFrEF who are referred for CRT implantation, also suggesting a course of treatment. Cardiac strain imaging results might be able to predict the chances of CRT being successful or not, allowing the interventional cardiologist to anticipate and plan for improved patient outcomes.

## Figures and Tables

**Table 1:** Patients’ characteristics before CRT implantation.

Parameter	ValueTitle
Age (years)	64 (53–70)
Sex: male female	37 (77%) 11 (23%)
Etiology of DCM: ischemic idiopathic	17 (35.4%) 31 (64.6%)
NYHA class: II III IV	20 (41.6%) 23 (47.9%) 5 (10.4%)
Associated diabetes mellitus Nondiabetics	20 (41.66%) 28 (58.33%)
Therapy: Beta-blockers ACE/ARB Sacubitril/valsartan Spironolactone Digoxin Furosemide	43 (89.58%) 22 (45.83%) 20 (41.66%) 38 (79.16%) 10 (20.83%) 47 (97.91%)

Legend: CRT—cardiac resynchronization therapy; DCM—dilated cardiomyopathy; NYHA—New York Heart Association; ACE—angiotensin-converting enzyme inhibitors; ARB—angiotensin receptor blockers.

**Table 2:** Echocardiographic parameters before CRT implantation.

**Electrocardiography**	
QRS (ms): initial post CRT implantation	160 (160–200) 130 (120–140)
Episodes of ventricular tachycardia	13 patients (27.08%)
Atrial fibrillation	9 patients (18.75%)
**Echocardiographic Parameters**	**Mean (min–max)**
LAV (mL)	101.5 (83.25–142)
LVEDV (mL)	236.5 (200–296.25)
LVEF (%—Simpson)	26 (20.25–30)
LV-GLS (%)	5.85 (3.82–7.2)
LV-PLS (%)	5 (2.125–8)
TAPSE cm	1.7 (1.5–1.97)
sPAP (mmHg)	35 (27–46.75)

Legend: CRT—cardiac resynchronization therapy; LAV—left atrial volume; LVEDD—left ventricular end-diastolic volume; LVEF—left ventricular ejection fraction; LV-GLS—left ventricular global longitudinal strain; LV-PLD—left ventricular postero-lateral strain; TAPSE—tricuspid annular plane systolic excursion; sPAP—systolic pulmonary artery pressure.

**Table 3:** Sensing and pacing parameters after CRT implantation.

CRT Parameter	Value
LV sensing (mV)	12 (9.5–17.5)
Pacing threshold LV (V)	3.5 (2.5–4)
Acute pacing threshold RV (V)	1 (0.625–1.5)
Acute sensing threshold RV (mV)	11 (8–12)
RA sensing (mV)	3.45 (2.5–3.8)

Legend: CRT—cardiac resynchronization therapy; LV—left ventricle; V—volt; RV—right ventricle.

**Table 4:** Correlations between the TTE parameters and LV sensing and pacing.

Parameter	LV Sensing			LV Pacing		
	r	95% CI	*p*	r	95% CI	*p*
LVEF (Simpson)	0.534	0.301; 0.716	˂0.001	−0.363	−0.608; −0.113	0.011
LV-GLS	0.650	0.407; 0.816	˂0.001	−0.567	−0.765; −0.317	˂0.001
LV-PLS	0.469	0.159; 0.726	0.001	−0.555	−0.793; −0.271	˂0.001
LAV (mL)	−0.574	−0.750; −0.333	˂0.001	0.385	0.122; 0.599	0.007
TAPSE (cm)	0.417	0.179; 0.616	0.003	−0.373	−0.597; −0.078	0.009
sPAP (mmHg)	−0.270	−0.497; −0.002	0.064	0.124	−0.169; 0.401	0.402

Legend: LV—left ventricle; LVEF—left ventricular ejection fraction; LV-GLS—left ventricular global longitudinal strain; LV-PLD—left ventricular postero-lateral strain; r—correlation coefficient; CI—confidence interval; *p*—statistical significance; Spearman correlation test. Statistically significant *p* < 0.05.

**Table 5:** Multivariate linear regression of independent factors for LV sensing.

Variable	β	Standard Error	*p*	95% CI for β
LVEDD (mL)	−0.016	0.005	0.006	−0.027; −0.005
LV-GLS (%)	1.046	0.148	<0.001	0.747; 0.145
LAV (mL)	0.023	0.007	0.003	−0.037; −0.008
Ventricular tachycardia	−2.187	0.936	0.025	−4.079; −0.296
NYHA class	−1.626	0.562	0.006	−2.761; −0.491
Diabetes Mellitus	−1.491	0.743	0.052	−2.994; 0.012

Legend: LAV—left atrial volume; LV-GLS—left ventricular global longitudinal strain; LVEDD—left ventricular end-diastolic volume; NYHA—New York Heart Association; β—regression coefficient; SE—standard error; *p*—statistical significance; CI—confidence interval; statistical method: multivariate stepwise linear regression (Akaike information criteria). Statistically significant *p* < 0.05.

**Table 6:** Multivariate linear regression of independent factors for LV pacing.

Variable	β	Standard Error	*p*	95% CI for β
LV-GLS	−0.116	0.057	0.049	−0.231; 0.000
LV-PLS	−0.095	0.038	0.015	−0.171; −0.019
TAPSE	−0.508	0.282	0.049	−1.078; 0.061
Diabetes Mellitus	0.504	0.210	0.021	0.080; 0.929

Legend: LV-GLS—left ventricular global longitudinal strain; LVEDD—left ventricular end-diastolic volume; LV-PLS—left ventricular postero-lateral strain; TAPSE—tricuspid annular plane systolic excursion; β—regression coefficient; SE—standard error; *p*—statistical significance; CI—confidence interval; statistical method: multivariate stepwise linear regression (Akaike information criteria). Statistically significant *p* < 0.05.

## Data Availability

Our data are available at https://doi.org/10.17632/w4536c7gpd.1.
